# Influence of Stimulus Layout and Social Presence on Deception-Related Eye Movements and Blinks in the Concealed Information Test

**DOI:** 10.3390/jemr19010021

**Published:** 2026-02-11

**Authors:** Valentin Foucher, Anke Huckauf

**Affiliations:** Faculty of Engineering, Computer Science, and Psychology, Experimental Psychology, Ulm University, 89081 Ulm, Germany; anke.huckauf@uni-ulm.de

**Keywords:** eye-tracking, gaze, eye movements, deception, conceal, lie, CIT, social presence

## Abstract

Over the past decades, eye movements and blinks have been integrated into Concealed Information Test (CIT) paradigms as indicators of deception. Recent findings suggested that fixation patterns in CITs depend on stimulus layout, particularly the distinction between sequential and simultaneous stimulus presentation. In addition, the impact of social presence on deceptive eye movements, critical for application of the CIT in real-world social settings, remains insufficiently examined. The present study addresses these issues through two experiments. In both, participants selected a card and had to reveal, conceal, or fake its value while all possible cards were displayed in pairs. Experiment 1 examined whether deceptive intentions could be differentiated using fixations and blinks, and extended previous findings on the effect of stimulus layout. Experiment 2 assessed the stability of deception-related eye movements and blinks across various levels of social presence (without, per video, being observed by a real person). Our findings replicate effects previously observed with simultaneous stimulus presentation of more cards, demonstrating how stimulus layout modulates deception-related eye movement patterns in CITs. The levels of social presence realised in this study did not significantly alter these patterns, indicating that deception-related eye movements and blinks in CITs remain stable under passive social presence.

## 1. Introduction

Deception is common in daily life. Studies report an average of two lies per day [1,2], and that children begin to lie at three to four years of age [3]. At the same time, human lie-detection performance is slightly above chance at roughly 54% [4]. Some domains require higher certainty, including criminal investigations and personnel selection. These demands have driven the search for methods and cues to deception that can support judgements of truth and lies [5]. Traditional deception paradigms such as the Comparative Question Test (CQT) rely on physiological reactions to detect covert lies, typically in response to oral questioning that would induce fear or stress [6,7], but has been criticised for weak theoretical bases, as false positives driven by strong emotional responses in innocent individuals may arise [8]. Deception was also associated with an increase in cognitive load as compared to being honest [5,9,10]. This load arises from different reasons, such as lie construction, truth suppression, memory load, or self-control [9]. The Concealed Information Test (CIT) was developed to uncover concealed knowledge by presenting a series of words or images while physiological or behavioural responses are recorded [11,12]. These measures are used to determine the recognition of privileged information, such as crime details [13] or familiar identities [14]. The CIT is based on the orienting response, which describes an individual’s reaction to a significant change in their environment [15], and which reflects the attention to essential information, even before a conscious decision is made. CIT measures can include galvanic skin response, breath and pulse rate, blood pressure [7], reaction times [16,17], P300 event-related potential (ERP) [18,19], and eye movements [20,21].

### 1.1. Eye Movements as Indicators of Deception

The use of eye-tracking in CITs is of interest because it can visualise attention and provide indirect measures of deception while remaining easy to implement, minimally intrusive, and affordable. Unlike autonomic physiological measures, eye movements originate from central nervous system processes, offering a distinct pathway for detecting deception [22,23]. Eye movements have been used to reveal deception across several studies over the last two decades: an increase in cognitive load was found to induce fewer but longer fixations [24]. Consistent with this, deception in CIT induced fewer but longer fixations on concealed items such as faces [14,25,26] or mock-crime material [27]. However, in the context of personality tests that involve multiple-response options, deception was found to increase fixation rates [28,29]. One potential reason, as suggested by the authors, is that deceiving these tests was cognitively easier, whereas responding honestly required a more thorough evaluation of the questions. But another reason could be the presence of multiple objects that may have influenced deceptive fixation patterns. Indeed, with identical concealment instructions, the stimulus layout has been reported to reverse the direction of the effects of deception on fixations, with sequential presentation induces fewer but longer fixations, while simultaneous presentation inducing more but shorter fixations compared to being honest [30]. Because the deceptive instructions imposed no difference in cognitive load across stimulus layouts, the observed differences in eye movement patterns are more plausibly attributable to strategic gaze modulation. Such modulation arises from high-order cognition but denotes deliberate, task-driven eye movement strategies employed to manage deceptive performance. Consequently, modulation of deception effects on eye movements by stimulus layout in CIT paradigms may account for conflicting findings in the literature and has important implications for the interpretation and design of CITs. Replication is therefore required to confirm this influence, which is examined in the present study.

Deception was also found to alter blink behaviour, as involuntary blinks can expose cognitive states such as mental and visual load or fatigue [31,32]. Indeed, concealment of information within CITs was found to produce a decrease in blink frequency [27,33,34,35]. A rebound increase in blink rate seems to emerge immediately after a lie [33,36]. Blinks also serve communicative functions during social interactions [37,38], indicating that social presence can modulate eye movement behaviours [39]. Indeed, faces are known to be salient stimuli, independent of task demands [40]. In visual search, passive social presence has been associated with fewer and shorter fixations in simple tasks, and more and longer fixations in complex tasks [41], although these effects have not been consistently replicated [42]. When viewing videoclips of a confederate only, participants directed less gaze towards this confederate when they believed her or him to be live compared to one believed to be pre-recorded [43,44]. These findings indicate that social presence influences eye movements and blinks across different contexts. Moreover, the sensation of being watched has been shown to reduce antisocial behaviours [43,45,46]. However, to our knowledge, no studies have examined the joint effects of social presence and deception on eye movements. Given that deception research ultimately targets real-world applications that inherently involve social dynamics [47], it is necessary to characterise how social presence may modulate deception-related eye movements.

### 1.2. Research Questions

Beyond laboratory settings, the CIT can be applied in real-world contexts to determine whether a suspect holds critical information, as is already the case in certain law-enforcement practices [47,48]. However, as highlighted in the literature, the validity of the CIT based on eye measures still requires further careful examination, particularly in relation to potential moderating factors such as the stimulus layout (Experiment 1) and the social presence (Experiment 2).

#### 1.2.1. Stimulus Layout

Previous work has shown that, despite identical concealment instructions, presenting either one or six stimuli at a time in a CIT led participants to adopt different deceptive strategies, resulting in opposite patterns of fixation numbers and duration [30]. Specifically, when cards were presented sequentially one at a time, concealment was associated with fewer and longer fixations, whereas when six cards were presented simultaneously, concealment produced more and shorter fixations. This pattern suggests that simultaneous object presentation elicits qualitatively different deceptive eye movements than sequential presentation, a factor that is critical for the interpretation of eye movements in CIT paradigms and may contribute to inconsistent findings. The goal of Experiment 1 was to further investigate this mechanism by implementing a card-based CIT in which participants selected a card and were instructed to reveal, conceal, or fake its value while pairs of cards were presented, thereby combining elements of both sequential and simultaneous stimuli presentation. If, as hypothesised by Foucher and Huckauf [30], presenting multiple stimuli is sufficient to reverse the direction of the fixation effect compared to single-stimulus presentation, the findings from Experiment 1 should align with those observed in the six-card simultaneous condition. Specifically, the concealing instruction is expected to elicit more but shorter fixations than the revealing or faking instructions when cards are presented in pairs. Such findings would provide additional evidence that multiple-object presentation modulates deception-related eye movement patterns in CIT paradigms as compared to single-object presentation.

#### 1.2.2. Social Presence

Most previous CIT studies have relied on screen-based stimuli. While this approach allows precise control over timing, randomisation, and stimulus properties, it lacks the social dynamics typically present in applied contexts, such as the presence of an examiner during the administration of a CIT in police investigations [47]. Some research indicated that social presence could influence eye movements [41,43]. Therefore, the extension of CITs to applied social contexts requires examination of the impact of social presence on deceptive eye movements. Consequently, the goal of Experiment 2 was to examine the interaction effect between deception and social presence on eye movements, and to evaluate the stability of eye movement indicators of deception in CITs in various social contexts. Using the same CIT experimental design as Experiment 1, two additional levels of social presence were compared: a video of a person observing the participant and the presence of a real observer, along with a control condition involving a neutral pixel-based picture. If social presence modulates deception-related eye movements, the video and real observer conditions should elicit eye movement patterns that differ from the control condition. Conversely, the absence of such differences would indicate that CIT eye measures are robust to passive social presence and remain applicable in contexts involving an examiner or investigator.

In summary, the present study pursued two objectives: Experiment 1 aimed to replicate the differentiation of deceptive intentions (faking, concealing, revealing) using fixations, to extend the findings using blinks, and to revisit the effects of sequential versus simultaneous card presentation, which have previously been shown to elicit different eye movement patterns [30]. Following successful replication of these findings, Experiment 2 introduced social presence as an additional factor to extend the ecological validity of the CIT by examining the reliability of deception-related eye movements at various levels of social presence (without, per video, with a real person).

## 2. Experiment 1

### 2.1. Materials and Methods

#### 2.1.1. Design

**Figure 1:**
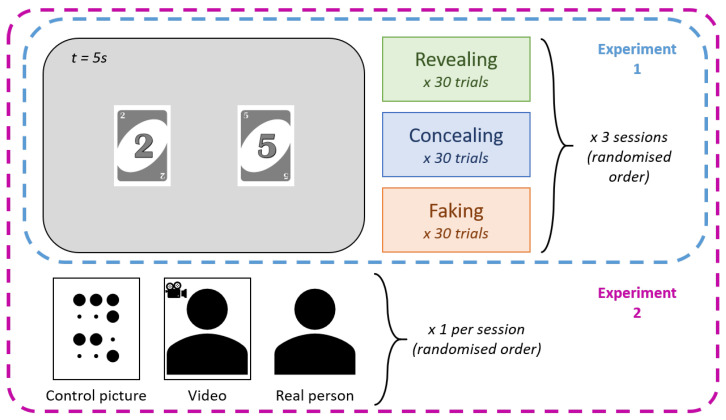
Structure of the experiments. Two cards were displayed simultaneously for 5 s, randomly selected from a set of six possible cards. Each deceptive instruction included the presentation of the 30 unique card pairs. Each session comprised the three deceptive instructions, resulting in a total of 270 trials per experiment. Experiment 2 followed the same procedure as Experiment 1, with the addition of social presence manipulation.

**Figure 2:**
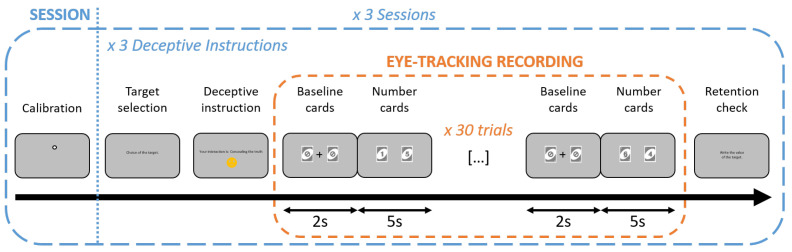
Structure of a session. Following calibration, participants selected a target and received the deceptive instruction (revealing, concealing, or faking). In the faking condition, a faked target was also selected. Cards were presented in pairs, alternating between baseline and number card presentations for 2 and 5 s, respectively, across the 30 trials. Participants then recall the target (and faked target when applicable) as a retention check. This sequence was repeated three times for each deceptive instruction. Three sessions were completed in total, with breaks between sessions.

**Figure 3:**
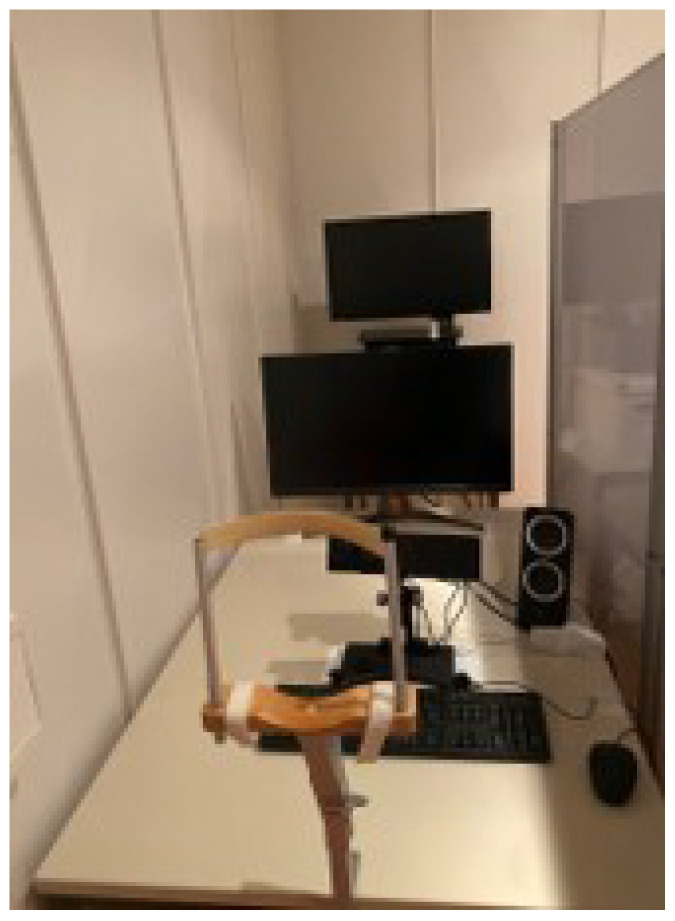
Laboratory setup. From bottom to top: chin rest, keyboard and mouse, Eyelink 1000, speaker (unused), experiment monitor, and social-presence monitor (only used in Experiment 2).

Participants were instructed to select one card from six UNO cards numbered one to six placed on a table. The selected number, hereafter referred to as the target, served as the critical item for the session. Participants were then instructed to either reveal, conceal, or fake the target while numeric copies of all six card values were presented on a monitor in pairs. All possible card pair combinations were presented for each deceptive instruction. Participants interacted with a smiley avatar just using their eyes, following one of three instructions: in the revealing condition, they attempted to make the avatar identify the target; in the concealing condition, they attempted to prevent the avatar from identifying the target; in the faking condition, they attempted to make the avatar identify a different card as the target, referred to as the faked target. Each session comprised the three deceptive instructions, and three sessions were completed in total, with a new target selected at the beginning of each interaction condition. In the faking condition, participants additionally selected a faked target that they intended the avatar to infer. For analytical clarity, this additional card was treated as a fourth level of deceptive instruction in the analyses. In Experiment 1, participants performed the task alone in the laboratory. The structure of the experimental task is illustrated in Figure 1, a session is illustrated in Figure 2, and the laboratory setup is shown in Figure 3.

#### 2.1.2. Procedure

Participants first received an introduction to the eye-tracking apparatus. They were then instructed on the experimental task, as described in the Section 2.1.1, and informed about the appearance of both the physical cards and their numeric on-screen replicas. The experiment was divided into three identical sessions to allow rest periods and reduce visual fatigue. Each session began with a calibration procedure enabling the eye tracker to associate gaze positions with screen locations. During calibration, participants visually tracked a dot that moved randomly from the centre to six different peripheral positions and then returned to the centre. The calibration phase lasted approximately one minute. Experimental trials started only after successful calibration.

The six physical number cards were placed side by side on the table in front of the participant throughout the experiment to provide a stable visual reference. Their order was randomised and reshuffled for each participant and for each session. Each interaction began with the selection of a new target card. Participants entered its value via the keyboard both prior to and following the on-screen presentation to ensure retention. In the faking condition, participants additionally entered the value of the card they intended the avatar to infer. Numeric card replicas were then presented on the screen in pairs. Neutral UNO baseline cards were displayed for 2 s, followed by the number cards for 5 s. Baseline and number card display alternated across the 30 trials. An intervening fixation cross that participants were required to fixate on was displayed between the two baseline cards. The order and spatial position of the number cards were pseudo-randomised. Across the 30 trials, each of the six numbers appeared 10 times, covering all possible left-right positioning and pairwise combinations. Following the retention check, a new target was selected, and a new deceptive instruction was given. Each session comprised the completion of the three deceptive instructions, with three sessions conducted in total. The order of the interaction instructions was randomised within each session. Each instruction included 30 trials, yielding 90 trials per session and 270 trials per participant.

Participants were instructed to interact only using their eyes to minimise bodily motion. While task objectives were specified, no strategy instructions were provided. Participants generated their own methods for conveying information, with the only constraints being to maintain gaze toward the screen and to remain physically still.

#### 2.1.3. Apparatus and Stimuli

Eye movements were recorded with an EyeLink 1000 from SR Research Ltd. (Kanata, ON, Canada). The setup consisted of a table supporting the monitors and the eye-tracker. Participants were seated at a distance of 57 cm from the screen in a room with low ambient light. To minimise movement artefacts, participants placed their head on a chin rest. Instructions were white-coloured and displayed on a grey background on a 27″ monitor with a resolution of 1920 × 1080 pixels and a 100 Hz refresh rate. Stimulus presentation and response collection were controlled by a custom programme written on Psychopy 2022.2.5 [49], running on Windows 10, 64-bits.

Real blue UNO cards were used for the initial target selection, chosen for their simple and unambiguous numeric design. To minimise luminance variations, standardised numeric UNO card replicas were used for on-screen presentation. These stimuli were rendered in grey and white using simple pixel discrimination, and their overall brightness was controlled across cards. Each numeric card had a size of 5.9 × 4.1 degrees of visual angle. The associated area of interest included an additional one-degree margin around each card to account for the spatial accuracy of the Eyelink 1000, resulting in a final region of 7.9 × 6.1 degrees of visual angle. A smiley avatar was used to represent the deceptive instruction but was not displayed during the recording phases. A green friendly smiley represented the revealing instruction, a yellow neutral smiley represented the concealing instruction, and an orange angry smiley represented the faking instruction.

#### 2.1.4. Participants

The study was advertised using the local university study management system (SONA Systems, Bethesda, MD, USA), and participants received hours’ credit as compensation for their participation. The requirements for participation were normal or corrected-to-normal vision and the absence of epileptic seizures. A total of 30 participants, including 23 females and 7 males, were recruited (M = 22.0, sd = 1.80). All participants were naïve to CIT paradigms prior to the study.

#### 2.1.5. Preprocessing

Gaze data were exported using EyeLink Data Viewer. Blink identification is based on missing pupil signals. Fixations shorter than 60 ms or longer than 5 s (card presentation duration) were excluded from the analysis [30]. Blinks shorter than 60 ms or longer than 700 ms were also excluded [50]. Fixations outside screen boundaries were removed, as calibration was limited to the display area. Gaze data was analysed for the period when the target was presented, and when the faked target was presented in the faking instruction. For group comparisons, the faking-target and faking-faked-target conditions were treated as distinct deceptive instructions in the statistical analysis. Consequently, four levels of deceptive instruction were analysed, although only three interaction instructions were explicitly given.

The analysis was conducted in R version 4.2.3 [51]. Fixations and blinks were analysed using linear mixed models with the “lme4” package [52]. F-values and p-values were estimated using Satterthwaite’s approximation via the “lmerTest” package [53]. Effect sizes were estimated using the “effectsize” package [54]. A post hoc power analysis was performed based on Lenth [55] methodology to investigate the reliability of our results and can be found in Appendix A Table A1.

An error rate was defined as the proportion of mismatches between the first target call (before card display) and the second call (after card presentation). The mean error rate across participants was 0.7%, with a maximum of 11.1% (one error over nine calls). No participant was excluded based on this error rate; however, trials containing mismatches were excluded from analysis.

To examine the influence of deceptive instructions on eye movements, mean fixation count, mean fixation duration, mean blink count, mean blink duration, and mean gaze transitions between cards were computed for each instruction within each session and then averaged per participant. Fixation and blink counts were normalised by dividing the number of events in a session by the number of cards actually presented for that instruction, yielding mean values per pair of cards that were comparable across instructions. Linear mixed models were then used to analyse these parameters according to the following formula: model=lmer(EyeParameter∼DeceptiveInstruction+(1|ParticipantID)).

### 2.2. Results and Discussion

#### Deceptive Instructions

**Figure 4:**
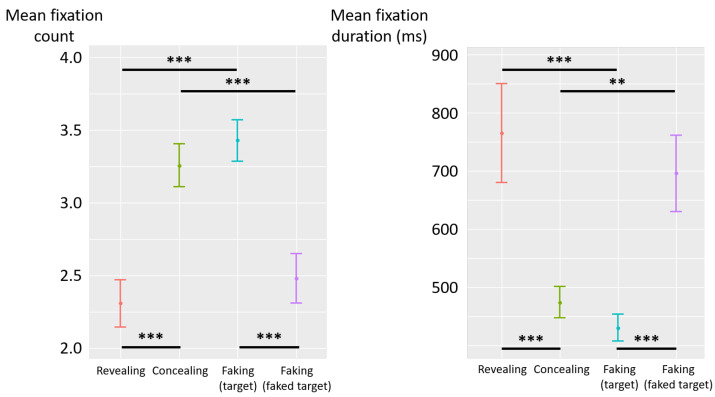
Experiment 1—mean fixation count (**left**) and mean fixation duration (milliseconds) (**right**) as a function of deceptive instructions. The error bars represent the standard error across participants. (**) *p* < 0.01, (***) *p* < 0.001.

The deceptive instructions had a significant influence on the count of fixations ($F\left(3,87\right)=19.08,\;p<0.001,\;\eta^{2}p=0.40$) (see Figure 4 left). Post hoc analyses revealed that the count of fixations was higher in the concealing condition than in the revealing (diff = 0.95, t(87) = 5.26, *p* < 0.001) and faking-faked-target (diff = 0.78, t(87) = 4.31, *p* < 0.001) conditions. Likewise, the count of fixations was higher in the faking-target condition than in the revealing (diff = 1.12, t(87) = 6.21, *p* < 0.001) and faking-faked-target (diff = 0.95, t(87) = 5.27, *p* < 0.001) conditions. No significant difference was observed between the concealing and faking-target conditions (diff = −0.17, t(87) = −0.95, *p* = 0.34), nor between revealing and faking-faked-target conditions (diff = −0.17, t(87) = −0.95, *p* = 0.35).

The deceptive instructions also had a significant influence on fixation duration ($F\left(3,87\right)=11.99,\;p<0.001,\;\eta^{2}p=0.29$) (see Figure 4 right). Post hoc analyses revealed that fixations were shorter in the concealing condition than in the revealing (diff = −291 ms, t(87) = −4.34, *p* < 0.001) and faking-faked-target (diff = −221 ms, t(87) = −3.30, *p* = 0.002) conditions. Likewise, fixations were shorter in the faking-target condition than in the revealing (diff = −335 ms, t(87) = −5.00, *p* < 0.001) and faking-faked-target (diff = −265 ms, t(87) = −3.96, *p* < 0.001) conditions. No significant difference was observed between the concealing and faking-target conditions (diff = 44 ms, t(87) = 0.66, *p* = 0.51), nor between revealing and faking-faked-target conditions (diff = 69 ms, t(87) = 1.04, *p* = 0.30).

**Figure 5:**
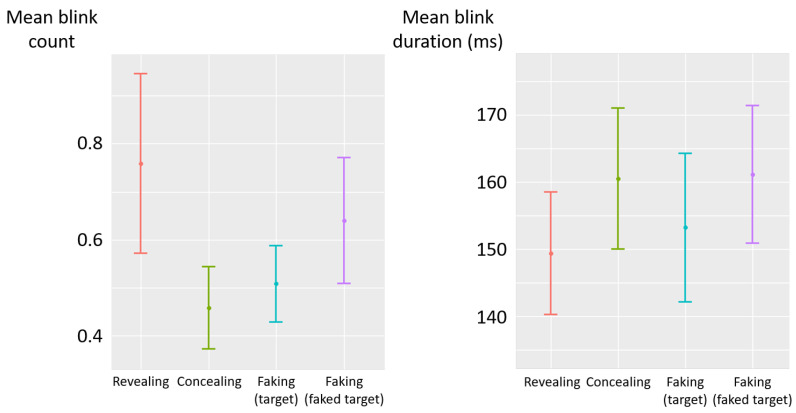
Experiment 1—mean blink count (**left**) and mean blink duration (milliseconds) (**right**) as a function of deceptive instructions. The error bars represent the standard error across participants.

There was no significant effect of the deceptive instructions on blink count ($F\left(3,85\right)=1.89,\;p=0.14,\;\eta^{2}p=0.06$) and duration ($F\left(3,84\right)=1.35,\;p=0.26,\;\eta^{2}p=0.05$) (see Figure 5).

**Figure 6:**
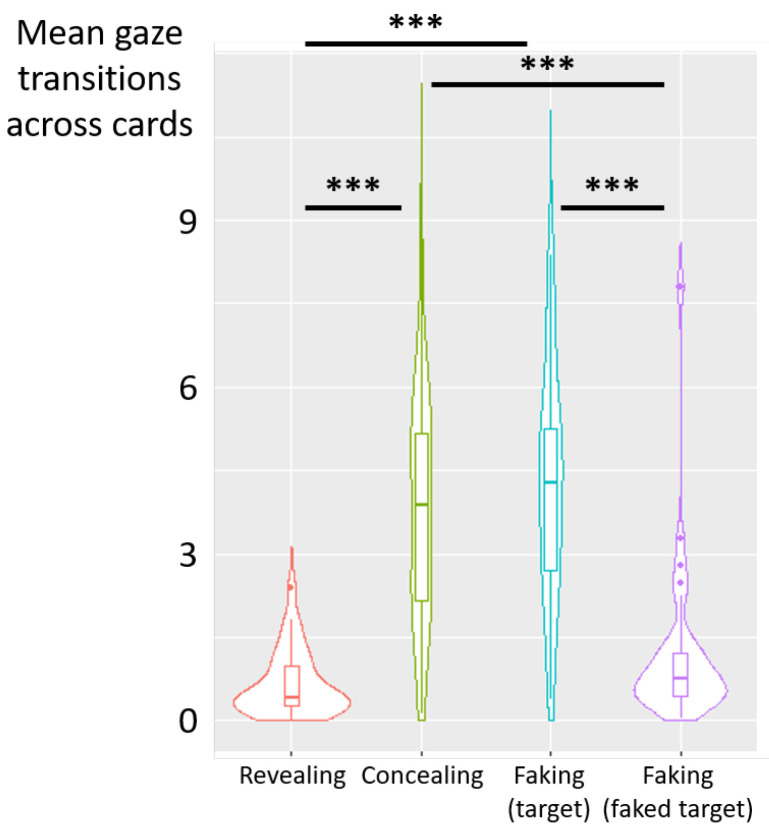
Experiment 1—violin plots showing the mean count of gaze transitions across cards as a function of deceptive instructions. The central marker indicates the median and the interquartile range across participants. (***) *p* < 0.001.

Deceptive instructions also had a significant influence on the count of gaze transitions between cards ($F\left(3,87\right)=44.05,\;p<0.001,\;\eta^{2}p=0.60$) (see Figure 6). Post hoc analyses revealed a higher count of transitions across cards in the concealing condition than in the revealing (diff = 3.09, t(87) = 8.09, *p* < 0.001) and the faking-faked-target (diff = 2.54, t(87) = 6.66, *p* < 0.001) conditions. Likewise, the count of transitions across cards was higher in the faking-target condition than in the revealing (diff = 3.58, t(87) = 9.37, *p* < 0.001) and faking-faked-target (diff = 3.03, t(87) = 7.94, *p* < 0.001) conditions. No significant difference was observed between the concealing and faking-target conditions (diff = −0.49, t(87) = −1.27, *p* = 0.21, nor between revealing and faking-faked-target conditions (diff = −0.55, t(87) = −1.43, *p* = 0.16).

All means and standard deviations are reported in Table 1.

To sum up, Experiment 1 assessed the impact of different forms of deception on eye movements using a CIT that displayed two cards at a time, combining sequential and simultaneous presentations, and tested whether stimulus layout modulated these effects. Target presentations produced more and shorter fixations, fewer blinks, and more gaze transitions between cards in the concealing and faking-target conditions as compared to the revealing and faking-faked-target conditions. Experiment 1 reproduced the pattern reported in the six-card display of Foucher and Huckauf [30], with concealment yielding more and shorter fixations, indicating an effect of stimulus layout on deceptive eye movements.

## 3. Experiment 2

### 3.1. Materials and Methods

The methods of Experiment 2 were the same as in Experiment 1 except for the differences described below.

#### 3.1.1. Design

As illustrated in Figure 1, in Experiment 2, the influence of social presence on deception and eye movements was investigated. Participants performed the same task (see Figure 2) while either a video of a person watching them, a pixel-scrambled image version of that video, or an actual person was present. The person was always a 21-year-old white man. The video consisted of a one-minute loop showing the experimenter looking toward the camera with subtle, natural head movements. The pixel image was generated by randomising the pixel distribution of a single video frame. Both the video and the pixel image were displayed on a second screen positioned behind the main task screen (see Figure 3). In the live condition, the second screen was removed, and the experimenter was at the same spatial position as he was when shown in the video. Each participant completed all three conditions in a randomised order. After completion of the experiment, participants rated their perception of social presence on a scale from 1 to 10.

#### 3.1.2. Apparatus and Stimuli

In Experiment 2, the second monitor displaying the picture and video was mounted on a table, with its centre positioned 160 cm from the participant. The experimenter’s eyes in the video were aligned with the centre of the screen. The monitor had a 23″ display with a 1980 × 1080 pixel resolution, and a 60 Hz refresh rate. In the live-observer condition, the second screen was removed and replaced by a chair positioned so that the experimenter’s eye level matched that of the person shown in the video condition. Floor markers ensured consistent placement of the table and chair across sessions and participants.

#### 3.1.3. Participants

Again, as in Experiment 1, the study was advertised using the local university study management system (SONA Systems), and participants received hours’ credit as compensation for their participation. The requirements for participation were normal or corrected-to-normal vision and the absence of epileptic seizures. In Experiment 2, 31 participants, including 19 females and 12 males, were recruited (M = 21.5, sd = 2.00). All participants were naïve to CIT paradigms prior to the study.

#### 3.1.4. Preprocessing

Experiment 2 had the same preprocessing steps as Experiment 1. The mean error rate across participants, defined as the proportion of mismatches between the first target call (before card display) and the second call (after card presentation), was 1.4% in Experiment 2, with a maximum of 11.1% (one error over nine calls). No participant was excluded based on this error rate; however, trials containing mismatches were excluded from analysis. Linear mixed models were used to analyse the effect of social presence on eye movements using the formula model=lmer(EyeParameter∼SocialPresence+(1|ParticipantID)). To assess its interaction with deceptive instructions, the following formula was applied: model=lmer(EyeParameter∼SocialPresence∗DeceptiveInstruction+(1|ParticipantID)). Bayes Factors (BF) were reported for the interaction between deceptive instruction and social presence using the “BayesFactor” package [56].

### 3.2. Results and Discussion

#### 3.2.1. Deceptive Instructions

**Figure 7:**
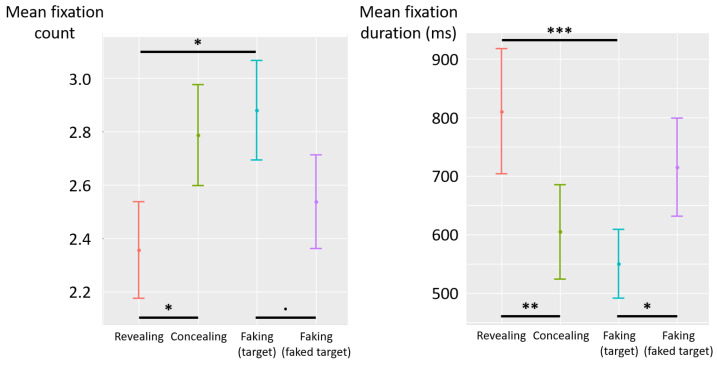
Experiment 2—mean fixation count (**left**) and mean fixation duration (milliseconds) (**right**) as a function of deceptive instructions. The error bars represent the standard error across participants. (.) *p* < 0.10, (*) *p* < 0.05, (**) *p* < 0.01, (***) *p* < 0.001.

The deceptive instructions had a significant influence on the count of fixations ($F\left(3,87\right)=3.33,\;p=0.02,\;\eta^{2}p=0.10$) (see Figure 7 left). Post hoc analyses revealed that the count of fixations was higher in the concealing condition than in the revealing condition (diff = 0.43, t(87) = 2.34, *p* = 0.02), and descriptively higher than in the faking-faked-target condition (diff = 0.25, t(87) = 1.35, *p* = 0.18). Similarly, the count of fixations in the faking-target condition was higher than in the revealing condition (diff = 0.52, t(87) = 2.84, *p* = 0.006), and marginally higher than in the faking-faked-target condition (diff = 0.34, t(87) = 1.85, *p* = 0.07). No significant difference was observed between the concealing and faking-target conditions (diff = −0.09, t(87) = −0.50, *p* = 0.62), nor between the revealing and faking-faked-target conditions (diff = −0.18, t(87) = −0.98, *p* = 0.33).

The deceptive instructions also had a significant influence on fixation duration ($F\left(3,87\right)=5.52,\;p=0.0021,\;\eta^{2}p=0.16$) (see Figure 7 right). Post hoc analyses revealed that fixations were shorter in the concealing condition than in the revealing condition (diff = −205 ms, t(87) = −2.95, *p* = 0.004) and descriptively shorter than in the faking-faked-target condition (diff = −110 ms, t(87) = −1.58, *p* = 0.12). Likewise, fixations were shorter in the faking-target condition than in the revealing (diff = −260 ms, t(87) = −3.73, *p* < 0.001) and faking-faked-target (diff = −165 ms, t(87) = −2.37, *p* = 0.02) conditions. No significant difference was observed between the concealing and faking-target conditions (diff = 54 ms, t(87) = 0.78, *p* = 0.44), nor between revealing and faking-faked-target conditions (diff = 95 ms, t(87) = 1.36, *p* = 0.18).

**Figure 8:**
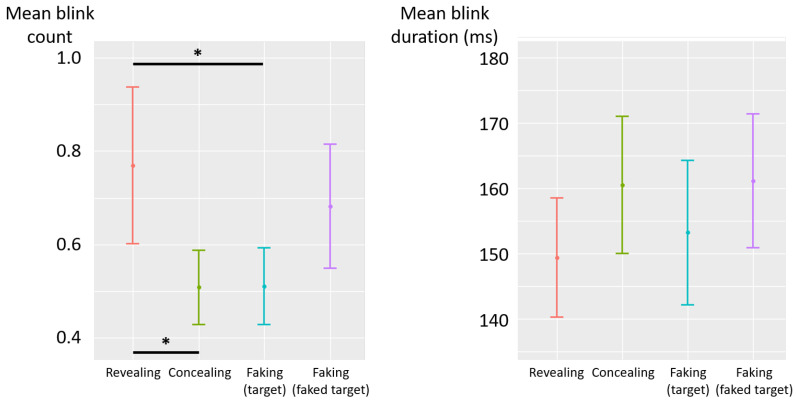
Experiment 2—mean blink count (**left**) and mean blink duration (milliseconds) (**right**) as a function of deceptive instructions. The error bars represent the standard error across participants. (*) *p* < 0.05.

The deceptive instructions had a significant influence on the count of blinks (F(3,87)=2.69, $p=0.05,\;\eta^{2}p=0.08$) (see Figure 8 left). Post hoc analyses revealed that the count of blinks was lower in the concealing condition than in the revealing condition (diff = −0.26, t(87) = −2.33, *p* = 0.02), and descriptively lower than in the faking-faked-target condition (diff = −0.17, t(87) = −1.55, *p* = 0.12). Similarly, the count of blinks in the faking-target condition was lower than in the revealing condition (diff = −0.26, t(87) = −2.31, *p* = 0.02), and descriptively lower than in the faking-faked-target condition (diff = −0.17, t(87) = −1.53, *p* = 0.13). No significant difference was observed between the concealing and faking-target conditions (diff = −0.002, t(87) = −0.02, *p* = 0.99), nor between revealing and faking-faked-target conditions (diff = 0.09, t(87) = 0.78, *p* = 0.44). There was no significant effect of the deceptive instructions on blink duration ($F\left(3,87\right)=1.75,\;p=0.16,\;\eta^{2}p=0.06$) (see Figure 8 right). A post hoc power analysis indicated that the effects of instruction on fixation and blink count may be underpowered and should be interpreted with caution (see Appendix A Table A1).

**Figure 9:**
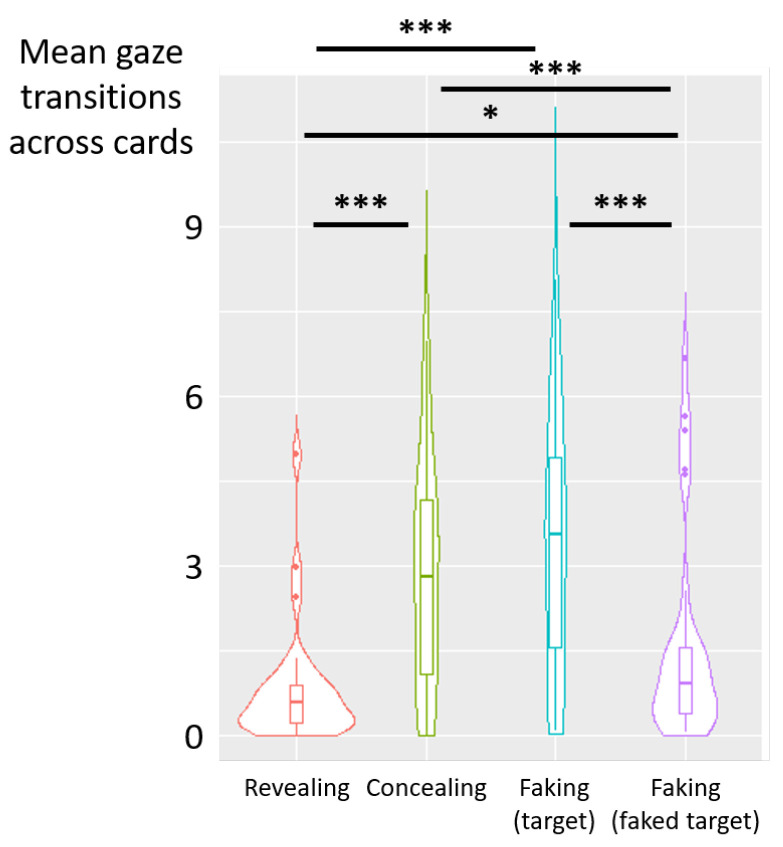
Experiment 2—violin plots showing the mean count of gaze transitions across cards as a function of deceptive instructions. The central marker indicates the median and the interquartile range across participants. (*) *p* < 0.05, (***) *p* < 0.001.

Deceptive instructions had a significant influence on the count of gaze transitions between cards ($F\left(3,87\right)=21.38,\;p<0.001,\;\eta^{2}p=0.42$) (see Figure 9). Post hoc analyses revealed a higher count of transitions across cards in the concealing condition than in the revealing (diff = 2.04, t(87) = 5.82, *p* < 0.001) and the faking-faked-target (diff = 1.25, t(87) = 3.56, *p* < 0.001) conditions. Likewise, the count of transitions across cards was higher in the faking-target condition than in the revealing (diff = 2.51, t(87) = 7.14, *p* < 0.001) and faking-faked-target (diff = 1.71, t(87) = 4.89, *p* < 0.001) conditions. No significant difference was observed between the concealing and faking-target conditions (diff = −0.46, t(87) = −1.32, *p* = 0.19, and a significant difference was found between the revealing and faking-faked-target conditions (diff = −0.79, t(87) = −2.26, *p* = 0.03).

All means and standard deviations are reported in Table 2.

#### 3.2.2. Social Presence

The manipulation check showed a significant effect of social presence on participants’ subjective distraction ($F\left(2,60\right)=37.31,\;p<0.001,\;\eta^{2}p=0.55$). Post hoc analyses indicated that the presence a real person was more distracting than both the video (diff = 1.16, t(60) = 3.14, *p* = 0.003) and the control picture (diff = 3.16, t(60) = 8.54, *p* < 0.001), and that the video was more distracting than the control picture (diff = 2.00, t(60) = 5.40, *p* < 0.001). However, social presence did not significantly affect fixation count (F(2,57)=0.84, p=0.44, $\eta^{2}p=0.03$), fixation duration ($F\left(2,57\right)=0.65,\;p=0.52,\;\eta^{2}p=0.02$), blink count ($F\left(2,57\right)=0.51,\;p=0.60,\;\eta^{2}p=0.02$), or blink duration (F(2,57)=0.66, p=0.52, $\eta^{2}p=0.02$).

**Figure 10:**
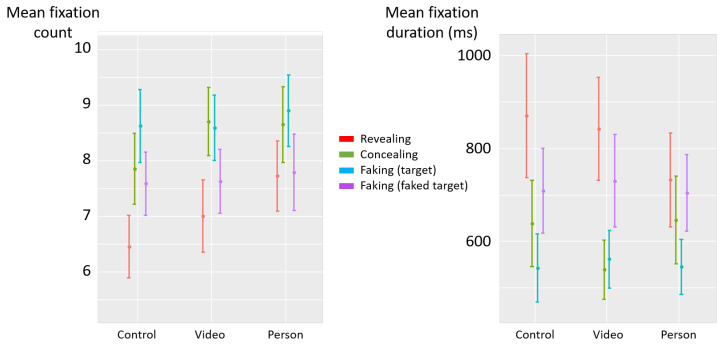
Experiment 2—mean fixation count (**left**) and mean fixation duration (milliseconds) (**right**) as a function of deceptive instructions and social presence. The error bars represent the standard error across participants.

**Figure 11:**
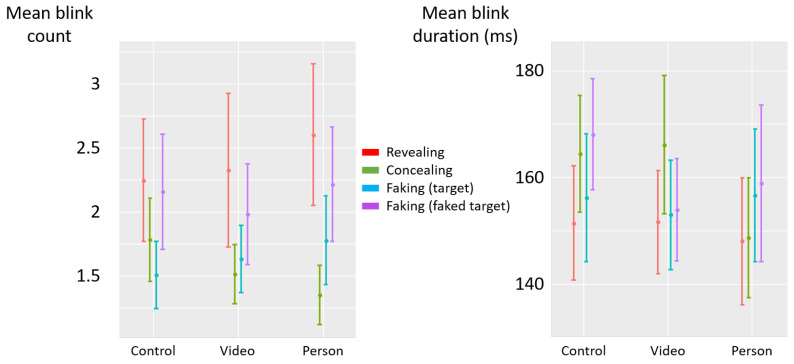
Experiment 2—mean blink count (**left**) and mean blink duration (milliseconds) (**right**) as a function of deceptive instructions and social presence. The error bars represent the standard error across participants.

There was no significant interaction effect of social presence and deception instructions on fixation count ($F\left(6,308\right)=0.39,\;p=0.88,\;\eta^{2}p=0.008,\;BF=82.9$) (Figure 10 left), fixation duration ($F\left(6,308\right)=0.71,\;p=0.64,\;\eta^{2}p=0.01,\;BF=74.5$) (Figure 10 right), blink count ($F\left(6,292\right)=0.28,\;p=0.94,\;\eta^{2}p=0.006,\;BF=83.8$) (Figure 11 left), or blink duration ($F\left(6,292\right)=0.24,\;p=0.96,\;\eta^{2}p=0.005,\;BF=80.0$) (Figure 11 right).

To sum up, Experiment 2 examined the stability of deception-related eye movements under social presence. The results replicate those of Experiment 1, with target presentation eliciting more and shorter fixations, fewer blinks, and more gaze transitions between cards in the concealing and faking-target conditions as compared to the revealing and faking-faked-target conditions, further confirming the influence of stimulus layout on these patterns. The manipulation of social presence did not significantly affect eye movements across deceptive conditions, indicating that deception effects on eye movements are stable to passive social presence.

## 4. General Discussion

The present study examined how deceptive intentions affect fixations and blinks in a card-based Concealed Information Test. Experiment 1 replicates that it is possible to differentiate concealing and faking on the basis of eye movements. In addition, it replicated the observations in studies using serial stimulus presentation and thus extends the findings on how stimulus layout determines the fixation effects in CIT. Experiment 2 also replicated the effects of deceptive intentions on eye movements and further demonstrated the stability of deception effects on eye movements under passive social presence, whether a video or a real person was observing the task.

Experiment 1 examined how different kinds of deception influence eye movements, using a design that combined sequential and simultaneous presentations by displaying two cards at a time to test whether the number of stimuli altered these effects. The presentation of the target elicited more and shorter fixations and descriptively fewer blinks in the concealing and faking-target conditions as compared to the revealing and faking-faked-target conditions. Also, concealing and faking-target conditions produced more gaze transitions between cards. The pattern indicates a deceptive strategy in which elevated eye movement activity served to obscure target recognition. These findings align with earlier work [30] proposing that different kinds of deception, notably concealing and faking, could be dissociated from eye movements. Faking appears to consist of a combination of a concealing phase when the target is shown, and a communicative phase when the faked target is presented. Concealment is reflected in fixation behaviour, whereas communicating during a CIT, whether truthful (revealing) or deceptive (faking-faked target), yields comparable fixation patterns. Furthermore, deception is typically linked to fewer but longer fixations, which can plausibly be attributed to increased cognitive load when lying as compared to being honest [14,25,26,27]. This might seemingly contradict studies reporting an increase in fixation count during deception; however, this occurred mainly in personality tests where lying has been suggested to be cognitively easier than responding honestly [28,29]. Previous work on CITs has shown that stimulus layout can reverse effect direction despite identical task requirements [30]. Presenting one card at a time sequentially versus six cards at a time simultaneously produced opposite fixation patterns, indicating that deceptive eye movements are subject to strategic gaze modulation. Unlike intrinsic deception-induced cognitive load, such as that arising from truth inhibition [9], this strategic modulation originates from high-order cognition and reflects deliberate eye movement strategies adapted to the stimulus configuration. Therefore, fixation behaviour in deception contexts reflects the combined influence of deception-induced cognitive load and strategic gaze modulation. Although stimulus layout was not manipulated as an independent factor in the present study, its influence was examined by combining sequential and simultaneous presentations within the same design, thereby testing whether previously reported layout effects would generalise to a two-card presentation. The fixation patterns observed in the six-card simultaneous display reported by Foucher and Huckauf [30] were replicated, with concealing producing more and shorter fixations than revealing or faking a target. This supports a functional distinction between single-object and multiple-objects presentations that should guide the design of new CIT protocols. Interestingly, studies reporting increased fixation counts during deception in personality tests also employed multiple response options [28,29]. Although these findings were attributed to deception being cognitively easier than truthful responding to the questions, the presence of multiple visual objects may also have contributed to the elevated fixation rates. These observations suggest that cognitive load and stimulus layout may jointly influence deception-related eye movements.

Experiment 2 assessed the stability of deception effects on eye movements under social presence. It replicated the findings of Experiment 1, with the presentation of the target eliciting more and shorter fixations, fewer blinks, and more gaze transitions between cards in the concealing and faking-target conditions as compared to the revealing and faking-faked-target conditions. This replication consolidates the evidence that eye movements can expose deception in CIT, and underscores the impact of the experimental design on effect patterns. The effect of deception on blinks, descriptive in Experiment 1, reached significance in Experiment 2. Both experiments had the same sample size and thus the same nominal power, which indicates that social presence amplified the blink response associated with deception. Blinks have been described as carrying a communicative function in social contexts where signalling understanding is relevant [37], which may account for their stronger modulation in Experiment 2. Interestingly, across all parameters examined in the present study, the number of gaze transitions between cards produced the largest effect size in both experiments. Although this measure is highly dependent on the visual design and spatial arrangement of the stimuli, it nonetheless appears to be a robust indicator of deception. As such, gaze-transition metrics may be particularly informative to increase certainty in detecting deception in paradigms that involve the simultaneous presentation of multiple objects, including applied CITs in forensic investigations [47] and questionnaire-based tasks with several response options in personnel screening [28,29]. However, the manipulation of social presence did not significantly alter eye movements, irrespective of deceptive instruction. Stronger social presence produced a descriptive increase in fixation count and a decrease in fixation duration as compared to the control condition, but there was no interaction effect with deceptive instructions. This indicates that deception generated the same eye movement patterns across social-presence levels. Although prior work in visual search tasks has indicated that gaze behaviours may be influenced by social presence [41], this effect has not always been reliable [42]. In the context of CIT, these findings highlight the stability of the effect of deception on eye movements and support the applicability of CITs beyond controlled laboratory settings [47]. Even though the study was not designed for direct comparison between experiments, the effect size concerning the effect of deception on fixations was smaller in Experiment 2 than in Experiment 1. The introduction of social presence in the laboratory setting may have induced a form of social facilitation [41], prompting participants to conceal or fake the target with greater care. This increased caution could in turn have reduced the magnitude of differences in eye movement patterns, resulting in smaller effect sizes.

### Limitations and Future Work

Several limitations can be considered from the present study. Both experiments were conducted in a university environment, yielding a sample with a homogeneous educational background and age. Prior work showed that personality traits could modulate deceptive behaviour [57], indicating that individual differences may affect the influence of deception on eye movements. The present findings require validation in a more diverse population. This study used a stationary Eyelink 1000 with a chin rest, preventing free head movements. Participants were instructed to perform the task solely with their eyes. This setup provided precise eye movement measurement and high data reliability, but also reduced ecological validity, as deception may unfold differently under less restrictive conditions.

A further limitation, typical in deception studies, concerns the instruction to lie. The design required participants to reveal, conceal, or fake the value of the target on demand to maintain experimental control, ensure balanced trial numbers and permit statistical comparison. However, this constrains generalisability, as real-world deception is typically self-initiated. Instructed deception also involves lower motivation because it lacks personal stakes, without consequences of being caught or incentives for remaining undetected. CITs have applications in high-stakes contexts, including criminal investigations, where they may be used to assess whether suspects or perpetrators are concealing critical information [58,59]. It is already implemented in real-world settings, notably within the Japanese police system [47,48]. The extent to which high-stakes situations, compared to low-stakes ones, affect an individual’s capacity to deceive or control involuntary responses remains uncertain and requires further empirical investigation [60]. Increased stakes may strengthen efforts to appear convincing, or they may elevate emotional load and amplify the effects observed under low-motivation conditions.

Because no effect of social presence on eye movements was observed in this study, a key limitation concerns the use of eye-tracking itself. Indeed, eye-trackers have been shown to create a feeling of being observed, which can alter participants’ behaviours, particularly in socially sensitive or norm-driven contexts [61]. Furthermore, the form of social presence implemented in the present study was passive, functioning as a background distractor rather than as an interacting social agent. Given that findings on passive social presence and eye movements are at least partially inconsistent [42], a more interactive social presence could produce stronger oculomotor modulation, and potentially lead to a different manifestation of deception in eye movement patterns.

## 5. Conclusions

The present study investigated whether eye movements and blink behaviour can reliably differentiate deceptive intentions in a card-based Concealed Information Test and whether these effects are modulated by stimulus layout and social presence. Across two experiments, distinct fixation and blink patterns were observed for revealing, concealing or faking the value of a card. Concealment was associated with a higher number of shorter fixations compared to revealing, while faking combined features of concealing the true target and revealing the faked one. The use of paired card presentation reproduced effects previously reported in simultaneous-display paradigms, providing evidence that stimulus layout, and more precisely the number of objects, systematically influences deception-related eye-movement patterns in the CIT. The second experiment extended the ecological relevance of these findings by introducing increasing levels of passive social presence. The presence of a control image, a video of an observer, or a real person did not significantly alter fixation or blink behaviours associated with deceptive intentions. These results demonstrate the stability of eye movements as markers of deception in CITs to passive social presence.

## Figures and Tables

**Table 1:** Experiment 1—means and standard deviations of the dependent variables across deceptive instructions.

Deceptive Instructions					
Parameter	Mean Fixation Count (sd)	Mean Fixation Duration (sd)	Mean Blink Count (sd)	Mean Blink Duration (sd)	Mean Transition Count (sd)
Revealing	2.31 (0.90)	765 ms (467 ms)	0.76 (1.03)	138 ms (38 ms)	0.66 (0.59)
Concealing	3.26 (0.81)	475 ms (148 ms)	0.46 (0.47)	150 ms (52 ms)	3.75 (2.04)
Faking-target	3.43 (0.79)	431 ms (126 ms)	0.51 (0.44)	135 ms (34 ms)	4.23 (2.06)
Faking-faked-target	2.48 (0.94)	696 ms (362 ms)	0.64 (0.72)	149 ms (61 ms)	1.20 (1.47)

**Table 2:** Experiment 2—means and standard deviations of the dependent variables across deceptive instructions and social presence.

Deceptive Instructions					
Parameter	Mean Fixation Count (sd)	Mean Fixation Duration (sd)	Mean Blink Count (sd)	Mean Blink Duration (sd)	Mean Transition Count (sd)
Revealing	2.36 (0.99)	810 ms (585 ms)	0.77 (0.92)	149 ms (50 ms)	0.82 (1.03)
Concealing	2.79 (1.04)	605 ms (437 ms)	0.51 (0.44)	161 ms (58 ms)	2.86 (1.95)
Faking-target	2.88 (1.02)	550 ms (321 ms)	0.51 (0.45)	153 ms (60 ms)	3.32 (2.24)
Faking-faked-target	2.54 (0.97)	715 ms (456 ms)	0.68 (0.73)	161 ms (56 ms)	1.61 (1.85)
**Social presence**					
Control	2.55 (0.86)	696 ms (494 ms)	0.64 (0.61)	159 ms (53 ms)	1.89 (1.15)
Video	2.63 (0.87)	661 ms (375 ms)	0.58 (0.59)	156 ms (54 ms)	2.14 (1.49)
Person	2.72 (0.97)	657 ms (423 ms)	0.63 (0.60)	152 ms (61 ms)	1.97 (1.55)

## Data Availability

Demographic and raw gaze data are stored securely on local hardware. Participants may request deletion of their data at any time. The aggregated data presented in the study are openly available in an OSF repository at https://osf.io/ufr75/overview?view_only=9dca3f10635a417bab18829b1d51ae87 (accessed on 15 December 2025).

## Abbreviations

The following abbreviations are used in this manuscript:

CIT	Concealed Information Test
M	Mean
sd	Standard Deviation
s	Seconds
ms	Milliseconds

## Appendix A. Post Hoc Power Analyses

**Table A1:** Post hoc power analysis based on Lenth [55] method for fixed-effects F tests, computed when p-values were under 0.05. A value of 1 rejects the null hypothesis. Legend: (N.S) Results were non-significant.

Deceptive Instructions					
Parameter	Fixation Count	Fixation Duration	Blink Count	Blink Duration	Transition Count
Experiment 1—Power	0.9952321	0.9881922	N.S	N.S	0.9995208
Experiment 2—Power	0.7673784	0.9507201	0.6246227	N.S	0.9963226
